# Interpreting population reach of a large, successful physical activity trial delivered through primary care

**DOI:** 10.1186/s12889-018-5034-4

**Published:** 2018-01-23

**Authors:** Sally M. Kerry, Katy E. Morgan, Elizabeth Limb, Derek G. Cook, Cheryl Furness, Iain Carey, Steve DeWilde, Christina R. Victor, Steve Iliffe, Peter Whincup, Michael Ussher, Ulf Ekelund, Julia Fox-Rushby, Judith Ibison, Tess Harris

**Affiliations:** 1 Pragmatic Clinical Trials Unit, Queen Mary’s University of London, London, SE 1 2AT UK; 2 Department of Medical Statistics, London School of Hygiene and Tropical Medicine, London, WC1E 7HT UK; 3 Population Health Research Institute, St George’s University of London, London, SW17 ORE UK; 4 Gerontology and Health Services Research Unit, Brunel University, London, UB8 3PH UK; 5 Research Department of Primary Care & Population Health, University College, London, NW3 2PF UK; 6 Department of Sport Medicine, Norwegian School of Sport Sciences, PO Box 4014, 0806 Oslo, Norway; 7 MRC Epidemiology Unit, University of Cambridge, Cambridge, CB2 OQQ UK; 8 Department of Public Health Sciences, Kings College London, London, SE1 1UL UK

**Keywords:** Physical activity, Randomised trials, Recruitment, Primary care, Non-participation

## Abstract

**Background:**

Failure to include socio-economically deprived or ethnic minority groups in physical activity (PA) trials may limit representativeness and could lead to implementation of interventions that then increase health inequalities. Randomised intervention trials often have low recruitment rates and rarely assess recruitment bias. A previous trial by the same team using similar methods recruited 30% of the eligible population but was in an affluent setting with few non-white residents and was limited to those over 60 years of age.

**Methods:**

PACE-UP is a large, effective, population-based walking trial in inactive 45-75 year-olds that recruited through seven London general practices. Anonymised practice demographic data were available for all those invited, enabling investigation of inequalities in trial recruitment. Non-participants were invited to complete a questionnaire.

**Results:**

From 10,927 postal invitations, 1150 (10.5%) completed baseline assessment. Participation rate ratios (95% CI), adjusted for age and gender as appropriate, were lower in men 0.59 (0.52, 0.67) than women, in those under 55 compared with those ≥65, 0.60 (0.51, 0.71), in the most deprived quintile compared with the least deprived 0.52 (0.39, 0.70) and in Asian individuals compared with whites 0.62 (0.50, 0.76). Black individuals were equally likely to participate as white individuals. Participation was also associated with having a co-morbidity or some degree of health limitation. The most common reasons for non-participation were considering themselves as being too active or lack of time.

**Conclusions:**

Conducting the trial in this diverse setting reduced overall response, with lower response in socio-economically deprived and Asian sub-groups. Trials with greater reach are likely to be more expensive in terms of recruitment and gains in generalizability need to be balanced with greater costs. Differential uptake of successful trial interventions may increase inequalities in PA levels and should be monitored.

**Trial registration:**

ISRCTN.com ISRCTN98538934. Registered 2nd March 2012.

## Background

Failure to include groups with lower physical activity (PA) levels in trials assessing the effectiveness of PA interventions, may increase health inequalities. The health benefits of PA are well established, but the numbers achieving recommended PA levels are generally low [1], with women, older people, those from socio-economically deprived areas and those of Asian ethnicity [1, 2] reporting lower levels. Although a number of walking intervention trials have been conducted [3], and recruitment rates of 30-40% [4–7], reported, some trials have much lower rates [8–10] and many population level PA interventions use advertising to recruit and cannot therefore estimate recruitment rates [11, 12]. The extent to which the results of such trials could be applied to those who might be offered the intervention in routine practice, sometimes referred to as an intervention’s ‘reach’ is unclear [13]. Reach is affected by the setting, participation rates and participant representativeness. Where participation rates are low [8–10], there may be systematic differences between those who participate and those who do not participate but who may represent a sizeable proportion of the population to whom the intervention could be appropriately offered.

Identifying trial recruitment inequalities is important to understand the limitations of the evidence, in terms of generalisability, and also to aid in planning studies. Failure to recruit is a major concern for research funders. Forty-five percent of trials funded by two UK funding agencies between 2002 and 2008 failed to recruit to target and required extensions [14]. The review found community and primary care trial recruitment similar to other settings, but did not investigate whether increased reach was associated with lower recruitment levels. Trials carried out in more ethnically diverse and socioeconomically deprived settings may be less likely to achieve recruitment targets.

The PACE-UP pedometer-based walking intervention trial recruited from primary care registers in seven south-west London practices [15]. The trial was successful as both intervention arms increased objectively measured physical activity levels at 12 months [16]. The population-based sampling frame provided an opportunity to assess differences in terms of age, gender, ethnicity and area-level deprivation between general practice (GP) patients who agreed to participate in the trial compared with those who did not, and between those who replied to the invitation letter compared with those who did not. We also compared health, lifestyle, education and social factors of those who agreed to participate with those who agreed to complete a questionnaire, but did not wish to participate.

## Methods

### The PACE-UP trial

The PACE-UP trial [15] recruited inactive 45-75 year olds registered at seven south-west London general practices and randomised 1023 people to one of three arms: an intervention arm designed to increase walking using pedometers, personalised walking plans and nurse consultations; a postal intervention arm (without nurse consultations); and a standard practice arm. Participants in the trial had to be able to walk outside and have no contraindications to increasing their PA levels.

Potentially eligible patients were identified using Read codes for medical conditions and local knowledge about care homes to exclude ineligible patients. The trial randomised households (one person living alone or two people with an age difference of less than 15 years if more than one eligible person in the household). Batches of approximately 400 potentially eligible individuals from randomly selected households were screened for exclusion criteria by general practitioners or practice nurses to avoid inviting patients with conditions that would exclude them from being offered the intervention in routine care, but which are not well recorded on Read codes (e.g., acute systemic illness such as pneumonia, unstable heart failure, unable to move about independently, psychotic illness). Some individuals will have already been screened out through READ codes. The remainder were invited to participate in the trial by letter, which included the trial information leaflet, a reply slip and stamped addressed envelope. Those willing to participate but who reported that they achieved, or were not sure if they achieved, recommended levels of at least 150 min weekly of at least moderate intensity physical activity [17], were telephoned to check their eligibility. If confirmed to be active they were excluded. One reminder letter was sent to those who did not reply. All trial information was written in English. For further details see the trial protocol [15].

### Data collection

The gender, age and Index of Multiple Deprivation (IMD) of all those invited were collected from GP records. IMD is an anonymised post-code deprivation measure [18]. To avoid the possibility of individuals being identified, aggregated practice-recorded ethnicity was exported in 10 year age-bands for all batches where everyone was mailed, less exclusions. We classified the practice recorded ethnicity into 4 categories, White (including ‘British or Mixed British’), Asian (including ‘Asian British’), Black (including ‘Carribbean’, ‘African’ and ‘Black British’) and Other.

Those not wanting to participate in the trial were asked if they would complete a shortened trial baseline questionnaire, including demographics, health, a primary care PA questionnaire (General Practice Physical Activity Questionnaire GPPAQ [19]), EQ-5D [20] and a question on reasons for not participating.

### Comparison groups

Individuals whose invitation letters were ‘returned-to-sender’ were excluded from analyses before calculating response rates. ‘Responders’ are defined as those who replied to the invitation letter, regardless of whether they wanted to take part or not. Individuals could respond by post, email or phone.

**Fig. 1:**
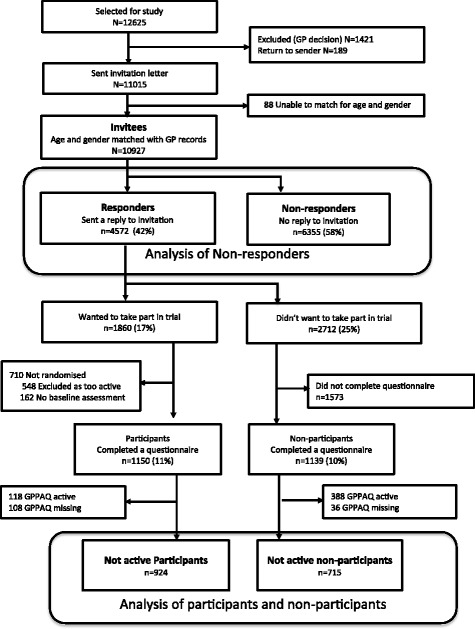
Flow chart to show the recruitment process in the PACE-UP trial. All percentages are out of all those whose age and gender were matched with GP records (10927)

‘Participants’ are those who completed baseline assessment, although not all were randomised as some provided inadequate objectively measured physical activity data. ‘Non- participants’ are those who completed a questionnaire but did not wish to participate in the trial (Fig. 1).

Since PACE-UP targeted inactive adults, participants who attended a baseline appointment were selected on the basis of their low PA levels. Non-participants were not selected in this way. In order to minimise selection bias, analysis of participants and non-participants was therefore restricted to those categorized as ‘not active’ according to GPPAQ, which was the only physical activity measure available on both groups.

### Statistical analysis

Age and gender standardised rates were used to compare IMD quintiles for responders. Similarly, gender standardised rates were used to compare age groups and age standardised rates to compare genders. The full population of invitees was used as a standard population throughout. No further analysis on non-responders was possible because they did not provide any questionnaire data on ethnicity or other factors.

Practice ethnicity data were extracted in 10-year age bands for 10,155 invitees from batches where everyone was mailed, effectively a random sample of the 11,015 invited. The proportion of patients belonging to each ethnicity category within age band and within practice was calculated and the number of invitees in each ethnicity in each practice and age-band was estimated. Overall, 1903 invitees had ethnicity recorded as ‘unknown’. These are assumed to be missing at random in the main results but sensitivity analyses were performed, assuming these were all white or all non-white. Age standardised participation rates for not active participants and non-participants completing questionnaires were calculated assuming that invitees gave the same ethnicity on the questionnaire as was recorded in their practice records. Participation rates by age, gender and IMD were calculated for not active participants versus not active non participants completing questionnaires, as in the analysis of responders.

Not active participants and non-participants completing the questionnaire were compared for additional demographic and social characteristics and health and lifestyle factors using logistic regression. All data came from questionnaires. Models were adjusted for clustering by practice and household by including fixed effects for practice and using robust standard errors for household.

## Results

Of 12,625 individuals selected for screening (see Fig. 1), 1421 (11.3%) were excluded by practice staff and 189 (1.5%) had invitation letters that were returned, as they had moved away; both of these groups were classified as ‘not invited’. In 44 households where one person refused the invitation and the other did not respond, it was impossible to match the response to individual invitees within household, so age and gender are unknown. These 88 people have been excluded from all further analyses. Of the remaining 10,927, 4572 (42%) responded to the invitation letter, mainly by post, and 1150 (11%) completed baseline assessments.

Of all invitees, 5229 (48%) were aged 45 to 54. Although all quintiles of deprivation were represented, only 7% were in the most deprived quintile. Response rates were higher in older people, women and those living in less deprived areas (Table 1). Since individual ethnicity was available only for the participants and non-participants who completed a questionnaire, it was not possible to estimate response rates by ethnicity for all responders.

**Table 1:** Responders to invitation letter by age, gender and Index of Multiple Deprivation (IMD)

	All invitees *N* = 10,927	Responders to invitation *N* = 4572		
	Number (%)	Number	Standardised percentage response ^a^ (95% CI)	Ratio of response rates (95% CI)
Age				
45-54 years	5229 (47.8)	1698	33.4 (32.1, 34.7)	0.57 (0.54,0.60)
55-64 years	3367 (30.8)	1535	46.2 (44.5, 47.9)	0.79 (0.76,0.84)
65-75 years	2331 (21.3)	1339	57.8 (55.8, 59.8)	1.0
Gender				
Female	5604 (51.3)	2638	46.7 (45.4, 48.0)	1.0
Male	5323 (48.7)	1934	36.8 (35.5, 38.1)	0.80 (0.76,0.84)
IMD national quintile ^b^				
1 Most deprived	712 (6.8)	207	29.5 (26.2, 32.8)	0.55 (0.50,0.61)
2	2768 (26.4)	995	36.1 (34.4, 37.9)	0.67 (0.63,0.72)
3	2960 (28.2)	1242	41.2 (39.8, 43.2)	0.77 (0.73,0.82)
4	2328 (22.2)	1060	45.6 (43.6, 47.5)	0.85 (0.80,0.90)
5 Least deprived	1711 (16.3)	914	53.4 (51.4, 56.0)	1.0

^a^ Age percentages standardised for gender, gender percentages standardised for age, IMD percentages standardised for age and gender. Percentages are of all those invited

^b^ 448 people are missing IMD, primarily due to certain postcode areas not being included in the look-up table

Although GPPAQ was not used to assess PA levels for trial inclusion, it was the only PA measure available for both participants and non-participants. 118 participants and 388 non-participants were classified as active by GPPAQ and 134 did not complete GPPAQ. These people were excluded from further analysis, leaving 924 participants and 715 non-participants.

Similar to response rates, participation rates were higher in older people, women and those living in less deprived areas (Table 2). Ethnicity was extracted from the practice for 10,155 invitees. Of these, 5991 were recorded as White (59%), 893 (9%) as Asian or British Asian and 915 (9%) as Black Caribbean, Black British or Black African. 1903 (18.7%) were recorded as ‘unknown’. The percentage ‘unknown’ varied by practice from 3% to 48%.

**Table 2:** Completion of baseline assessment and questionnaires in participants and non-participants who are not active on GPPAQ by age, gender, Index of Multiple Deprivation (IMD) and ethnicity

	All invitees *N* = 10,927		Participants *n* = 924		Non-participants *n* = 715		
	N	N	Standardised completion rate^a^ (95% CI)	Ratio of completion rates	N	Standardised completion rate^a^ (95% CI)	Ratio of completion rates (95% CI)
Age							
45-54 years	5229	331	6.4 (5.7, 7.1)	0.60 (0.51,0.71)	238	4.6 (4.0, 5.1)	0.41 (0.34,0.49)
55-64 years	3367	342	10.1 (9.1, 11.1)	0.94 (0.81,1.10)	213	6.3 (5.5, 7.1)	0.56 (0.47,0.67)
65-74 years	2331	251	10.8 (9.4,11.9)	1.0	264	11.2 (10.0, 12.6)	1.0
Gender							
Female	5604	597	10.6 (9.8, 11.4)	1.0	408	7.2 (6.5, 7.9)	1.0
Male	5323	327	6.2 (5.6,6.9)	0.59 (0.52, 0.67)	307	5.9 (5.2, 6.5)	0.82 (0.71, 0.94)
IMD national quintile ^b^							
1Most deprived	712	40	5.5 (3.8, 7.2)	0.52 (0.39,0.70)	31	4.5 (3.0,6.0)	0.51 (0.37, 0.70)
2	2768	183	6.7 (5.7,7.6)	0.63 (0.52, 0.78)	128	4.6 (3.8, 5.4)	0.52 (0.41,0.66)
3	2960	288	9.6 (8.6,10.7)	0.92 (0.77,1.10)	213	7.1 (6.2, 8.0)	0.80 (0.65, 0.98)
4	2328	206	8.8 (7.7, 10.0)	0.84 (0.69, 1.02)	172	7.4 (6.3, 8.4)	0.83 (0.67, 1.03)
5Least deprived	1711	179	10.5 (9.1,11.9)	1.0	150	8.9 (7.5, 10.2)	1.0
Ethnicity							
White	8129^c^	709	8.7 (8.1, 9.3)	1.0	638	7.9 (7.3, 8.4)	1.0
Asian	1131^c^	61	5.4 (4.1, 6.7)	0.62 (0.50, 0.76)	27	2.4 (1.5, 3.3)	0.31 (0.24, 0.38)
Black	1084^c^	90	8.5 (6.7, 10.2)	0.97 (0.79, 1.20)	20	1.9 (1.1, 2.8)	0.24 (0.19, 0.31)
Other	583^c^	22	3.8 (2.2,5.4)	0.44 (0.33,0.59)	20	3.9 (2.2,5.6)	0.59 (0.36 0.68)

^a^ Age percentages standardised for gender, gender and ethnicity percentages standardised for age, IMD percentages standardised for age and gender. Percentages are of all those invited

^b^ 448 people are missing IMD, primarily due to certain postcode areas not being included in the look-up table

^c^ Number of invitees estimated from practice summary data

Of the White invitees 709 (8.7%) agreed to participate in the trial and were not active and a further 638 (7.9%) completed a non-participant questionnaire and were not active. Both Asian and Black invitees had very low non-participant questionnaire completion (2.4% and 1.9%) but black invitees were as willing to participate as white invitees (8.5% v 8.7%), while only 5.4% of Asians participated. Sensitivity analyses assuming that all ethnicities recorded as ‘unknown’ were white or non-white showed similar results, and the same patterns were also seen in practices with nearly complete ethnicity coding.

Compared with non-participants providing questionnaire data, participants were more likely to be working part-time, to be married or living with a partner, and to have finished their education between 17 and 18 years (Table 3). Participation was associated with recent primary care contact and with some degree of health problems (general health, long standing illness and co-morbidities), although those more severely affected were less likely to participate (Table 3). This is consistent with EQ-5D (health-related quality of life) domains, where participants were more likely to have problems with pain and mobility but less likely to have problems with self-care, which is likely to indicate greater disability.

**Table 3:** Participants and non-participants who completed questionnaires and were not active on GPPAQ: demographics, and health and lifestyle factors

	Participants with baseline information *N* = 924^a^ Number (%)	Non-participants who completed a questionnaire *N* = 715^a^ Number (%)	OR for participation adjusted for clustering ^b^ (95% CI)	OR for participation adjusted for clustering, age and gender (95% CI)
Demographic factors				
Household structure				
Invited as couple	393 (42.3)	314 (43.9)	0.98 (0.79, 1.21)	0.99 (0.79,1.23)
Current marital status				
Married/Living together as a couple	595 (65.8)	439 (62.5)	1.20 (0.96, 1.49)	1.25 (1.01, 1.56)*
Age finished full-time education				
16 years or under	238 (26.4)	246 35.6)	0.64 (0.49, 0.83)	0.67 (0.51, 0.87)
17 or 18 years	204 (22.6)	122 (16.2)	1.39 (1.10, 1.76)	1.23 (0.93,1.64)
19 years or over	334 (48.3)	334 (48.3)	1.0**	1.0**
Employment status				
Full time	334 (37.1)	248 (35.4)	1.0***	1.0**
Part time	175 (19.4)	83 (11.8)	1.60 (1.17, 2.19)	1.57 (1.13, 2.18)
Retired	274 (30.4)	269 (38.4)	0.77 (0.60, 0.99)	0.87 (0.63, 1.21)
Other	118 (13.1)	101 (14.4)	0.87 (0.64, 1.19)	0.85 (0.62, 1.17)
Home owner	734 (82.7)	587 (84.2)	0.92 (0.69, 1.23)	0.91 (0.68,1.21)
Health and lifestyle factors				
Contact with GP or nurse in last 3 months	591 (65.4)	409 (59.3)	1.31 (1.61,1.06)*	1.34 (1.09,1.65)**
Current smoker				
Yes	74 (8.4)	62 (9.0)	0.90 (0.63, 1.29)	0.87 (0.60, 1.24)
General health level				
Very good/good	727 (81.0)	579 (84.0)	1.0*	1.0*
Fair	154 (17.2)	88 (12.8)	1.34 (1.01, 1.79)	1.40 (1.05, 1.86)
Poor/Very poor	16 (1.8)	22 (3.1)	0.54 (0.28, 1.04)	0.56 (0.29, 1.09)
Limiting long-standing illness				
Yes, a lot	24 (2.7)	46 (6.7)	0.40 (0.24, 0.66)	0.41 (0.24, 0.70)
Yes, a little	194 (21.7)	113 (16.4)	1.35 (1.04, 1.77)	1.40 (1.07, 1.84)
No	678 (75.7)	528 (76.9)	1.0***	1.0***
Comorbidities				
One or more	568 (58.6)	401 (41.4)	1.23 (1.01,1.51)*	1.29 (1.05, 1.59)*
Number of different medications taken per day				
One or more	517 (57.6)	384 (55.5)	1.07 (0.87, 1.31)	1.17 (0.95, 1.46)
EQ-5D				
Mobility				
Some problems	202 (22.4)	122 (17.4)	1.36 (1.05, 1.76)*	1.44 (1.10, 1.87)**
Self-care				
Some problems	23 (2.6)	31 (4.4)	0.53 (0.30, 0.93)*	0.56 (0.32, 0.99)*
Usual activities				
Some problems	163 (18.3)	121 (17.2)	1.06 (0.81, 1.38)	1.09 (0.83, 1.43)
Pain/discomfort				
Some problems	522 (58.0)	326 (46.4)	1.61 (1.31, 1.97)***	1.62 (1.32, 2.00)***
Anxiety/depression				
Some problems	247 (27.8)	169 (24.0)	1.20 (0.96, 1.52)	1.19 (0.94, 1.50)
Health factors relating to exercise				
Balance problems				
Yes	106 (11.7)	64 (9.3)	1.26 (0.91, 1.76)	1.27 (0.90, 1.78)
Number of falls in past year				
Once or more	157 (17.5)	123 (18.0)	0.97 (0.74, 1.26)	0.98 (0.75, 1.27)
Walking pace				
Brisk/Fast	256 (27.9)	342 (48.2)	0.42 (0.34, 0.51)***	0.39 (0.32, 0.49)***
Someone to walk with				
Sometimes / Often / Always	791 (87.2)	600 (84.2)	1.25 (0.93, 1.69)	1.20 (0.88, 1.63)

^a^ Total number in each group. Some questions have missing data

^b^ ORs are from models with fixed effects for practice and robust standard errors for clustering by household

^*^p < 0.05; **p < 0.01; ***p < 0.001 from Wald test *p*-value for inclusion of the variable in the logistic model, used to assess significance of inclusion of categorical variables with more than two categories

Participants were less likely to walk fast, but there was no statistically significant association between participation and having someone to walk with (Table 3), having balance problems or falling.

Insufficient time was given by 45% (*n* = 327) of all invitees and 60% of 45-54 year olds as a reason for non-participation (Table 4). Even though those classified on GPPAQ as active were excluded from this analysis, 45% of invitees gave being sufficiently active as a reason, more commonly cited by men and those in less deprived areas. There were no clear trends with ethnicity but the number of non-participants in ethnic minority groups was small. Less commonly, 152 (21%) answered they could not or were not interested in (122, 17%) increasing their PA. Randomisation was only cited as a reason for non-participation by 88 (12%) of respondents.

**Table 4:** Reasons for non-participation by age, gender, Index of Multiple Deprivation (IMD) and ethnicity for not active non participants

		Do not have enough time n = 327		Already sufficiently active *n* = 325	
	Total in category	N (%)	Odds ratio (95% CI)	N (%)	Odds ratio (95% CI)
Age					
45-54 years	231	144 (60.5)	1.00***	93 (39.1)	1.00
55-64 years	213	99 (46.5)	0.55 (0.37, 0.81)	100 (47.0)	1.35 (0.92, 1.97))
65-74 years	264	84 (31.8)	0.29 (0.20, 0.43)	132 (50.0)	1.55 (1.08, 2.22)
Gender					
Female	408	196 (48.0)	1.0	171 (41.9)	1.0*
Male	307	131 (42.7)	0.82 (0.61, 1.11)	154 (50.2)	1.41 (1.06,1.89)
IMD national quintile					
1Most deprived	31	9 (29.0)	0.62 (0.22, 1.81)	8 (25.8)	0.20 (0.07, 0.55)
2	128	62 (48.4)	1.33 (0.69, 2.57)	55 (43.0)	0.58 (0.30,1.10)
3	213	102 (47.9)	1.37 (0.82, 2.31)	85 (39.9)	0.47 (0.28,0.79)
4	172	80 (46.5)	1.24 (0.76,2.01)	94 (54.7)	1.05 (0.65,1.69)
5Least deprived	150	63 (42.0)	1.0	77 (51.3)	1.0**
Ethnicity					
White	638	293 (45.9)	1.0	302 (47.3)	1.0
Asian	27	15 (55.6)	1.37 (0.58, 3.23)	9 (33.3)	0.67 (0.29,1.53)
Black	20	6 (30.0)	0.39 (0.14,1.10)	6 (30.0)	0.52,0.19, 1.41)
Other	20	9 (45.0)	0.98 (0.36,2.67)	6 (30.0)	0.51 (0.18,1.45)

*p < 0.05; **p < 0.01; ***p < 0.001 from Wald test *p*-value for inclusion of the variable in the logistic model, used to assess significance of inclusion of categorical variables with more than two categories

## Discussion

### Summary of findings

The PACE-UP trial recruited 11% of patients aged 45 to 75 invited by post by their practice. Those replying were older, more likely to be female and from less deprived postcodes. Participants in the trial who completed a baseline assessment and were not classified as active by questionnaire, were also more likely to be older, female, and from less deprived postcodes compared with non-active non-participants. Asian patients were less likely to participate. Participation was associated with having some comorbidity or some degree of health impairment, and having had recent primary care contact. Insufficient time and perceiving themselves as being already physically active were common reasons for non-participation, even though we only included those who were classified by GPPAQ as not active.

### Comparison with previous work

A systematic review of 47 walking intervention studies [3] showed recruitment methods and participation rates were poorly reported. Participation rates could only be calculated for 5/25 randomised controlled trials. We recruited by post to reduce practice staff burden and to obtain response rate data. Primary care uses postal invitations for other preventive activities, making this a pragmatic approach [21]. Other primary care walking interventions using postal invitations [8, 22–24] had similar response rates of 10 to 20%. Dubbert [4, 5] had higher rates (37% and 39%) but recruited additionally through routine primary care visits and patients were over 60 with on average 3.8 co-morbidities. We have shown that older age, having some comorbidity and recent health professional contact were associated with increased trial participation. Our previous trial [7] used similar recruitment strategies to PACE-UP and had a 30% recruitment rate, but was limited to over 60 year olds, was conducted in an affluent setting with few non-white residents and did not exclude those reporting they were active.

Non-responders were followed up with one reminder letter, but due to data protection constraints we could not telephone non-responders. Although only 1% of invitation letters were returned to sender, this may underestimate those not receiving the letter, as we did not used registered post. A previous London study using registered post found 26% of letters were not delivered [25]. Warner [24] found active refusal rates to be low but about 30% of eligible patients could not be contacted.

Most recruitment studies focus on recruitment methods [9, 26, 27]. In this study we compared participation rates for different groups within one primary care study using postal invitations. We have previously explored this in the PACE-Lift trial [28] setting, but PACE-UP is larger, with greater diversity, allowing ethnicity and deprivation effects to be explored comprehensively. We have already published findings from interviews to explore reasons for non-participation [29] in PACE-UP.

Our finding of greater participation in women, older people and those in affluent areas are supported by other studies [28, 29]. Although Attwood [30] found no association with deprivation or ethnicity this was in a highly deprived area with few non-white patients.

Among Asian patients, our response rate was similar to postal invitations in the PODOSA trial (5.2%) where community based approaches [31], through partnership with local South Asian groups were found to be more effective. Wilbur found social networking the most effective method for recruiting African American women from low income areas [27].

### Strengths and limitations

PACE-UP is a large trial recruiting from a clearly defined invited population, based on GP lists, enabling us to assess the potential reach of the intervention in terms of age, gender and deprivation. Our estimate of 11% participation may be an underestimate of the true rate, particularly in areas of high mobility.

Although based on limited data, the PACE-UP trial offers a rare opportunity to examine demographic differences between participants and non-participants. We were able to estimate participation within different ethnicities using pooled data from the practices. However, we were not able to match at an individual level and some participants may have categorised themselves in a different ethnic group to that on the GP register. Ethnicity was also poorly recorded in some practices and we needed to make assumptions about whether those with ‘unknown’ ethnicity were similar to those with recorded ethnicity. In a sensitivity analysis, even under extreme assumptions, the same ethnic variations persisted. It is possible we have underestimated the response rates in White people compared to other groups because in our estimation of the number of white invitees we classified as ‘White’ those classified as ‘British or Mixed British’; the practice data did not distinguish between ‘British’ and ‘Mixed British’. However comparing with the area census data (Wandsworth in 2011) [32] we believe any effect would be small.

The trial excluded individuals who self-reported being active, but the non-participants were not selected in this way. Our analysis attempted to mitigate this difference by restricting analysis to all those who self-reported as not being active using the same question. However, some residual bias may remain.

One possible limitation is that we did not attempt to compare different recruitment methods. Our main focus was to reach our recruitment target of 993 randomised patients in 1 year and we had insufficient resources to design a study within a trial [33] to compare different recruitment strategies. We chose to use postal recruitment which had been successful in our previous trial and we planned conservatively so that we had a large enough pool of invitees to achieve our target even with a low response rate. Copeland et al. [9] tried a number of strategies to boost recruitment in addition to postal invitation. These included asking GPs to recruit during consultations, and asking community leaders, health trainers and champions to distribute recruitment packs but these were all unsuccessful and all participants were recruited postally. Recruitment strategies based on advertising or community groups could be compared in terms of research effort required but would not allow characteristics of participants and non-participants to be compared; one of the key strengths of this study.

### Implications

NICE guidelines [34] conclude that more research is needed to determine which interventions are effective and cost-effective in increasing activity levels among lower socio-economic and high risk groups, and that there is little evidence on differential effects of interventions. In our trial those groups for which more evidence is required tended to be those with the lowest recruitment rates, such as Asians and those in more deprived areas [2]. It has been suggested [35] that specific cultural groups may respond better to interventions directly targeted at their needs, rather than to universal interventions. Reasons for non-participation often related to individuals not wanting to increase activity or feeling that they were sufficiently active. It is likely such resistance will similarly apply to any intervention roll out and may apply more widely to other public health interventions. Low participation rates mean policy makers should be cautious about the intervention’s potential reach and the possibility that it could increase activity inequalities, but is not a reason not to implement an intervention shown to be effective in 11% [16] of the population. We were successful in recruiting older people, women and those with co-morbidities or some degree of health limitation. These groups have lower PA levels and are likely to benefit more from increased physical activity. However, those with more severe disability, fallers, and those with a fear of falling were not over-represented, indicating a rational choice by individuals.

Only 12% of non-participants cited randomisation as a factor for not participating, while 45% cited time constraints. The nurse intervention required three additional visits to the practice on top of the three data collection visits, which may deter working people or those with childcare and other commitments. However, PACE-UP showed that both the nurse and postal groups performed similarly at the main 12 month outcome [16]. An intervention offering pedometers with brief advice, without the need to provide research data, may be more acceptable.

Both PACE-Lift and PACE-UP recruited to target, achieved follow up rates of over 90% and demonstrated the interventions were effective in increasing physical activity [16, 24]. However, considerably more research effort was required per randomised participant in PACE-UP compared with PACE-Lift, due to lower uptake. In spite of the effort, we still had limited power to investigate ethnic and socio-economic subgroups.

## Conclusions

Participation in an effective physical activity trial among adults and older adults in a socially and ethnically diverse population was only 11% with lower rates in more deprived and Asian subgroups, limiting the trial’s ability to investigate differential effects in these important subgroups. Trials with greater reach are likely to be more expensive in terms of recruitment and gains in generalizability need to be balanced with greater costs. Differential uptake of interventions found to be successful in trials may increase inequalities in PA levels and should be monitored.

## Abbreviations

GPPAQ: General Practice Physical Activity Questionnaire
IMD: Index of Multiple Deprivation
PA: Physical activity
